# Associations between Cardiorespiratory Fitness and Health-Related Quality of Life

**DOI:** 10.1186/1477-7525-7-47

**Published:** 2009-05-28

**Authors:** Robert A Sloan, Susumu S Sawada, Corby K Martin, Timothy Church, Steven N Blair

**Affiliations:** 1 Health Promotion Center, United States Naval Hospital Yokosuka, Kanagawa, Japan; 2 Health Promotion Center, Tokyo Gas Co., Ltd, Tokyo, Japan; 3 Pennington Biomedical Research Center, Baton Rouge, Louisiana, USA; 4 University of South Carolina, Columbia, South Carolina, USA

## Abstract

**Background:**

There is limited data examining the association between cardiorespiratory fitness (CRF) and health related quality of life (HRQOL) in healthy young adults. We examined the association between CRF and the HRQOL Physical Component Summary (PCS) and Mental Component Summary (MCS) scores in apparently healthy males in the United States Navy.

**Methods:**

A total of 709 males (18–49 yr) performed a submaximal exercise test and HRQOL assessment (SF-12v2™) between 2004–2006. CRF level was classified into fourths depending on age distribution with the lowest fitness quartile serving as the referent group. PCS and MCS scores ≥ 50 were defined as above the norm. Logistic regression was used to obtain odds ratios (OR) and 95% confidence intervals (CI). ResultsThe age-standardized prevalence of above the norm scores was lowest in the referent CRF quartile, PCS 56.6% and MCS 45.1%. After adjusting for age, systolic blood pressure, body mass index, smoking habit, alcohol habit and using the lowest CRF group as the reference, the OR (95% CI) for PCS scores above the norm across the fitness quartiles (P < 0.003 for trend) were 1.51(0.94–2.41), 2.24(1.29–3.90), and 2.44 (1.30–4.57); For MCS the OR (95% CI) were across the fitness quartiles (P trend < 0.001) 2.03(1.27–3.24), 4.53(2.60–7.90), 3.59(1.95–6.60).

**Conclusion:**

Among males in the United States Navy relative higher levels of CRF are associated with higher levels of HRQOL.

## Introduction

The Centers for Disease Control and Prevention and the Agency for Healthcare Research and Quality consider the surveillance of mental and physical health to be imperative in understanding health-related quality of life (HRQOL) and its impact on increasing the quality and years of healthy life, eliminating health disparities, and predicting future medical health care costs [1,2]. Higher levels of cardiorespiratory fitness (CRF) have been shown to be associated with higher levels of HRQOL in the older and chronically diseased populations [3,4]. However, there is a dearth of evidence on the association of CRF level and HRQOL in apparently healthy young adults. A recent systematic review highlighted the public health importance of better understanding the relation between physical activity and HRQOL in the general adult population [5]. Specifically, the authors noted that cross-sectional studies demonstrated positive associations between physical activity and HRQOL. The review presented minimal evidence for the relationship of objectively measured CRF and the mental and physical health components of HRQOL. It is well accepted that the primary marker for habitual physical activity is objectively measured CRF [6]. Therefore, this observational study sought to evaluate the association between CRF level and the physical and mental components of HRQOL in apparently healthy young males.

## Methods

### Data Source

The data were obtained from the Naval Hospital Yokosuka, Japan, Health Promotion Center health fitness assessment (HFA) database. A component of the HFA was the Short Form 12 version 2 (SF-12v2™) questionnaire [7]. Trained U.S. Navy medical personnel record the data during each initial HFA. The HFA data for this observational study was approved as exempt research by the Navy Medical Research Center, San Diego Institutional Review Board.

### Participants

For these analysis, data were examined from the review of 1127 HFA records of male United States Navy service members who were self-referred or referred by their primary care manager for health related lifestyle management counseling during 2004–2006. Participants included in the study were 18–49 years old, had been in the Navy for at least 6 months, graduated high school, and were considered apparently healthy documented by medical record review. Exclusion criteria included any participant with a history of a chronic condition or disease, psychotropic medication, cholesterol medication, blood pressure medication, an inability to reach 85% of maximal heart rate during the submaximal graded exercise test, or were missing any data required for the analysis. Any participant who reported smoking on the morning of the HFA was excluded from the database to avoid inaccurate prediction of metabolic equivalent (MET) capacity from the treadmill test. After review of the database 709 (62.9% of the original sample), apparently healthy participants were eligible for inclusion. Ethnicity and education level beyond high school were not recorded. All participants had a review of their medical record when they arrived for the HFA, which is primarily used to note any relative or absolute contraindications prior to exercise testing [8]. The SF-12v2™ was completed along with a generic self-report health risk appraisal that included tobacco and alcohol use questions. All medications were verified and documented prior to resting blood pressure and cardiorespiratory fitness testing. Resting heart rate and ausculatory blood pressure were completed per the JNC 7 guidelines [9].

Assessment and definition of health-related quality of life. HRQOL is defined as the perception of overall satisfaction with life and involves the measurement of functional status in the domains of physical, cognitive, emotional, and social health, and is a fundamental assessment in understanding the health status of a population [10]. The SF-12v2™ is a generic health status instrument that assesses HRQOL by asking twelve Likert scale questions that measure eight domains: physical function, role-physical, bodily pain, general health, vitality, social functioning, role-emotional, and mental health [7]. These eight domains are summarized into physical (PCS) and mental (MCS) component summary scales via established norm based scoring (NBS) algorithms [11]. Once the scores are transformed, the general population has a mean of 50 and a standard deviation of 10. Therefore when compared to the general population, HRQOL is considered to be below the norm if PCS or MCS scores are calculated to be below 50.

Assessment and definition of cardiorespiratory fitness. Each submaximal CRF test began within 30 minutes post completion of the SF-12v2™ questionnaire. A modified Balke protocol started at 88 m·mm^-1^(3.3 mph) and began with a grade of 0% and after every 3 minutes the grade was increased by 3% until the participant reached 85% of their age predicted heart rate max. Maximal MET level was estimated by using the method of extrapolation to the age predicted maximal heart rate [8]. ACSM guidelines for submaximal exercise testing were followed throughout the course of each test and all tests were administered by ACSM certified personnel [8]. We categorized participants into quartiles depending on age-specific (18–24, 25–29, 30–34, 35–39, 40–45, and 45–49) distributions of estimated maximal MET level.

### Data Analysis

Estimated maximal MET capacity levels were divided into quartiles with the lowest quartile serving as the referent group. Descriptive statistics were examined across fitness quartiles. Logistic regression was used to calculate odds ratios (OR) and 95% confidence intervals (CI) with the lowest CRF quartile as the reference category. OR were adjusted for age, body mass index, systolic blood pressure, current smoking (yes or no), and current alcohol intake (< 14 or ≥ 14 drinks per week). The Statistical Package for Social Science (version 12.0) was used for statistical analysis (SPSS, Inc., Chicago, Illinois, USA). All probability values of P < 0.05 were considered statistically significant.

## Results

Table 1 depicts baseline characteristics of the subjects (N = 709) according to CRF. Men in the referent quartile (9.7 ± 1.1 METS) had mean PCS and MCS scores below 50. Table 2 depicts the OR and CI for above the norm PCS and MCS scores by age-specific CRF levels, with the lowest CRF level as the referent. We observed significant positive trends across CRF categories for the prevalence and OR of PCS and MCS scores above the median. After multivariate adjustment for several potential confounding variables, low CRF was associated with low PCS and MCS scores. Pearson correlation coefficients between MCS vs. MET and PCS vs. MET were r = 0.078 (p = 0.037) and r = 0.269 (p < 0.001) respectively.

**Table 1:** Baseline characteristics of men according to cardiorespiratory fitness levels

	**Cardiorespiratory fitness levels, quartiles**				
Characteristics	All men	Q_1 _(Referent)	Q_2_	Q_3_	Q_4 _(High)
n	709	175	178	180	176
Age (years)	31.6 ± 7.4	31.7 ± 7.4	31.6 ± 7.4	31.7 ± 7.4	31.5 ± 7.4
PCS	52.3 ± 7.3	49.6 ± 7.8	52.0 ± 7.2	53.4 ± 7.0	54.3 ± 6.4
MCS	51.3 ± 8.3	48.0 ± 8.8	50.7 ± 8.0	53.5 ± 7.4	52.8 ± 7.7
Body mass index (kg·m^-2^)	28.7 ± 4.3	32.9 ± 3.8	29.6 ± 3.1	27.2 ± 2.9	25.3 ± 3.1
Systolic blood pressure (mmHg)	124.1 ± 11.6	126.1 ± 11.4	125.6 ± 12.6	122.2 ± 11.1	122.7 ± 10.8
Diastolic blood pressure (mmHg)	74.9 ± 10.3	76.7 ± 10.4	76.4 ± 10.3	73.6 ± 10.4	72.8 ± 9.6
Current smokers (%)	23.1	27.4	25.3	21.1	18.8
Current drinkers (%)	2.7	2.9	2.2	3.3	2.3

PCS, Physical Component Summary; MCS, Mental Component Summary.

*Data are means ± SD, unless otherwise specified.

**Table 2:** Odds ratios above the norm for MCS and PCS according to cardiorespiratory fitness levels

	**Cardiorespiratory fitness levels, quartiles**				
	Q_1 _(Referent)	Q_2_	Q_3_	Q_4 _(High)	*P *for trend
n	175	178	180	176	
PCS					
Prevalence of PCS Scores ≥ 50	56.6	69.1	78.3	81.3	
Age-adjusted OR (95% CI)	1.00	1.72 (1.11–2.67)	2.81 (1.76–4.48)	3.35 (2.06–5.44)	< 0.001
Multivariate OR* (95% CI)	1.00	1.51 (0.94–2.41)	2.24 (1.29–3.90)	2.44 (1.30–4.57)	0.003
MCS					
Prevalence of MCS Scores ≥ 50	45.1	61.8	77.2	73.3	
Age-adjusted OR (95% CI)	1.00	2.06 (1.33–3.19)	4.45 (2.77–7.13)	3.62 (2.28–5.75)	< 0.001
Multivariate OR* (95% CI)	1.00	2.03 (1.27–3.24)	4.53 (2.60–7.90)	3.59 (1.95–6.60)	0.001

OR, odds ratio; CI, confidence interval.

*Adjusted for age, BMI, systolic blood pressure, alcohol, habit, smoking habit.

## Discussion

This observational study investigated the association between CRF and HRQOL in young, apparently healthy men in the U.S. Navy. Our results suggest that there is a positive relationship between the level of CRF and the mental and physical health components of HRQOL. To the best of our knowledge, this is the first study to evaluate the associations between objectively measured CRF and HRQOL in apparently healthy young men.

Brown et al. conducted a large (N = 175,850) cross-sectional study on self-reported physical activity and HRQOL using the 2001 Behavioral Risk Factor Surveillance System (BRFSS) database [12]. A graded dose response relationship was established for physical activity and HRQOL that supports the current physical activity recommendations by the American College of Sports Medicine and the American Heart Association [13]. Puetz reviewed the epidemiological evidence for the dose response relationship between physical activity and vitality [14]. Vitality is one component for HRQOL that is used in the determination of MCS and PCS scoring. His review demonstrated considerable evidence that the greatest risk for low vitality is associated with sedentary lifestyle. Even though subjective measures of physical activity were used, the Brown et al. study and Puetz's review provide strong evidence for the relationship of physical activity and HRQOL.

Our study is one of only two studies that have demonstrated a relationship between objectively measured CRF level and HRQOL [5]. Galper et al. [15] reported a similar positive relation between CRF and mental health by measuring emotional well-being in 5230 men from the Aerobics Center Longitudinal Study. Although the authors used a different tool to quantify HRQOL, emotional well-being is considered to be a component of HRQOL.

The present study had several strengths. First, CRF and BMI were measured objectively. In comparison with self-report methods of estimated physical activity, CRF is a more objective measure [6]. It has also been found that self-report methods of BMI are influenced by under reporting for weight and over reporting for height [16]. Second, we used a well established, valid, and reliable measure of HRQOL that utilized norm-based scoring methodology. Norm based scoring allows for comparison between other studies that evaluate PCS and MCS regardless of the SF version used and avoids the ceiling effect sometimes seen in the eight SF-36 v2™ domains [5,11]. The third unique strength of this study was that the population we observed was young and apparently healthy U.S. Navy personnel.

The primary limitation of this study is that it was a cross-sectional design from which we cannot determine a direct cause and effect relationship. Generalizibility of this study may be limited because it was conducted only with males in the U.S. Navy. However, it should be noted, that baseline HRQOL norms for the healthy U.S. population with no chronic conditions, for PCS and MCS are 54.3 ± 6.2 and 52.3 ± 7.9, respectively [11]. These scores are only slightly higher from our observed baseline PCS and MCS mean scores of 52.3 ± 7.3 and 51.3 ± 8.3 respectively. Furthermore, there appears to be some similarity between our PCS and MCS baseline means and the U.S. Military whereby the Millennium cohort study of (N = 77047) unadjusted means for PCS and MCS norms were 53.4 and 52.8 respectively [17]. Although education level beyond high school may be considered a confounding variable, the Millennium cohort study indicted minimal to no significant differences in education level for adjusted PCS and MCS means [17]. The referral process (self or primary care) may be considered a possible limitation influencing motivation. The final limitation of our study is that submaximal testing was used to estimate maximal MET level. However, in its scientific statement on the Assessment of Functional Capacity in Clinical and Research Settings, the American Heart Association remarked that submaximal testing is a valid method to assess CRF [18].

## Conclusion

In conclusion the results of this study suggest that low CRF is associated with lower HRQL in apparently healthy young men. Future studies should focus on apparently healthy women along with prospective and clinical designs that demonstrate cause and effect. Also because of the independent association found in our study and other studies [4,12,14,15] between physical activity or CRF and HRQOL, studies investigating the role of BMI level on HRQOL should not exclude measures of physical activity or CRF.

## Competing interests

The authors declare that they have no competing interests.

## Authors' contributions

RS designed and conducted the study and drafted the manuscript. RS performed the data management, RA and SS contributed to the statistical analysis. RS and SS participated in the study design. RS, SS, CM, TC and SB contributed substantially to the manuscript. All authors read an approved the final manuscript.
